# Supplementary material for: Novel anti-repression mechanism of H-NS proteins by a phage protein
Source: Nucleic Acids Res. 2021 Sep 14;49(18):10770–84. doi: 10.1093/nar/gkab793 (PMC8501957; doi:10.1093/nar/gkab793)
Supplement: gkab793_Supplemental_File [file gkab793_supplemental_file.pdf]

Supplementary Information

**Novel anti-repression mechanism of H-NS proteins by a phage protein.**

Fredj Ben Bdira[1,2]*, Amanda M. Erkelens[1,2#], Liang Qin[1,2#], Alexander N. Volkov[3,4], Andrew M. Lippa[5], Nicholas Bowring[1,2], Aimee L. Boyle[1], Marcellus Ubbink[1], Simon L. Dove[5] & Remus T. Dame[1,2]*.

[1] Department of Macromolecular Biochemistry, Leiden Institute of Chemistry, Einsteinweg 55, 2333 CC Leiden, the Netherlands.
[2] Centre for Microbial Cell Biology, Leiden University, Einsteinweg 55, 2333CC Leiden, the Netherlands.
[3] VIB-VUB Structural Biology Research Center, Pleinlaan 2, 1050 Brussels, Belgium.
[4] Jean Jeener NMR Centre, VUB, Pleinlaan 2, 1050 Brussels, Belgium.
[5] Boston Children's Hospital, Division of Infectious Diseases, Harvard Medical School, Boston, MA 02115, USA.

[#]These authors contributed equally to this work

[*]Corresponding authors:
Fredj Ben Bdira: fredjbdira@gmail.com
Remus T. Dame: rtdame@chem.leidenuniv.nl

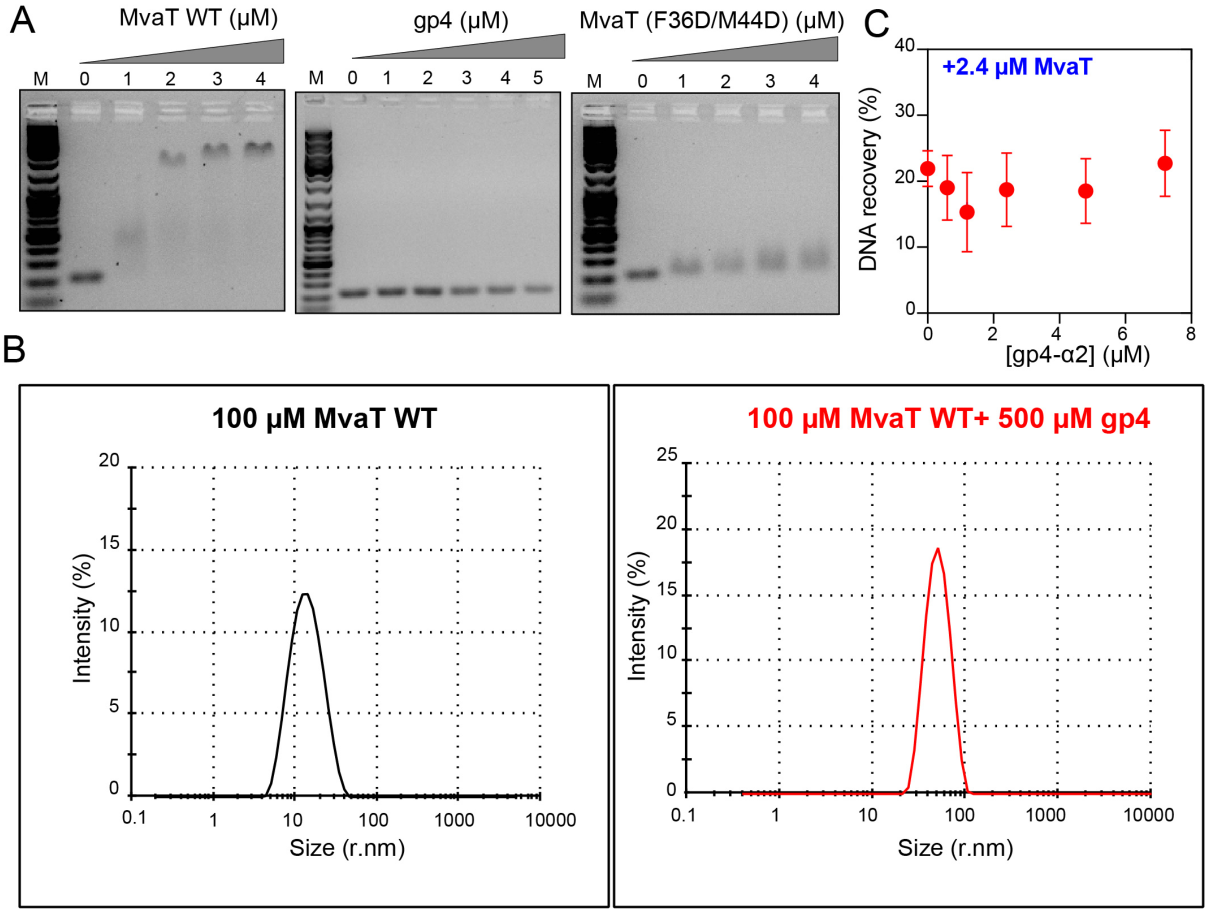


**Figure S1**: (A) Electrophoretic mobility shift assay (EMSA) of a 200bp DNA substrate at different concentrations of MvaT wild type (left panel), gp4 (middle panel) and MvaT F36D/M44D (right panel). (B) Dynamic light scattering profiles of 100 µM of MvaT wild type in the absence (left panel) and presence of 500 µM gp4 (right panel) in 20mM Tris-HCl, pH 8, 300 mM KCl. Note the increase in particle size of MvaT upon complex formation with gp4. (C) Effect of the gp4 C-terminal helix (residues 30-46) on MvaT DNA bridging activity.

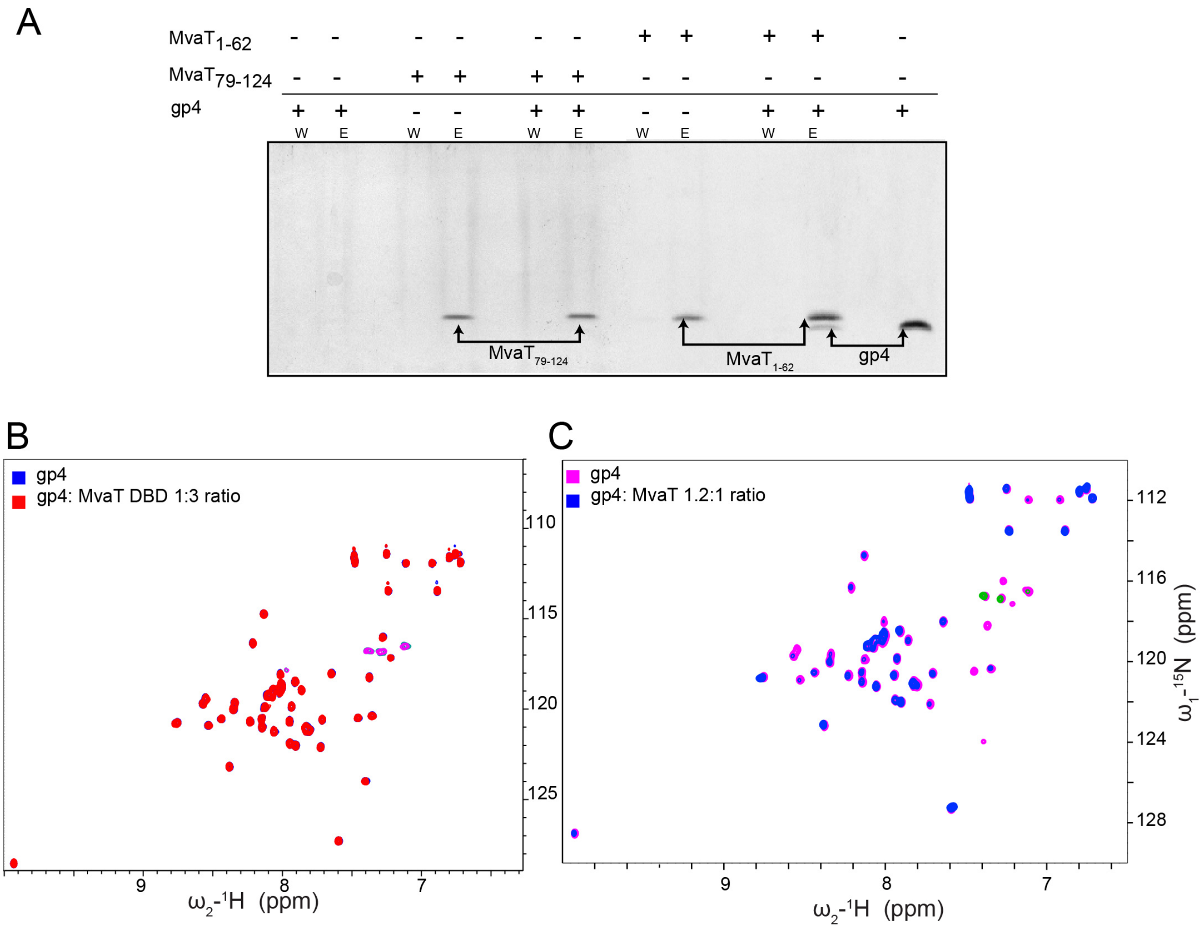


**Figure S2:** (A) His-tag pull down assay of MvaT truncated domains (NTD is 1-62 and DBD is 79-124) in the presence of gp4 analyzed by Tricine gel electrophoresis. W and E are for the flow through of the washing and elution steps, respectively (see M&M). (B) Overlay between $^{15}$N gp4 HSQC spectra in the absence (blue) and presence of unlabeled MvaT DBD at a gp4:DBD 1:3 molar ratio. Magenta and green are for the folded peaks of gp4 arginine side chains. (C) Overlay between $^{15}$N gp4 HSQC spectra in the absence (magenta) and presence (blue) of MvaT$_2$ at a gp4:MvaT 1.2:1 ratio. Light green is for the folded peaks of gp4 arginine side chains.

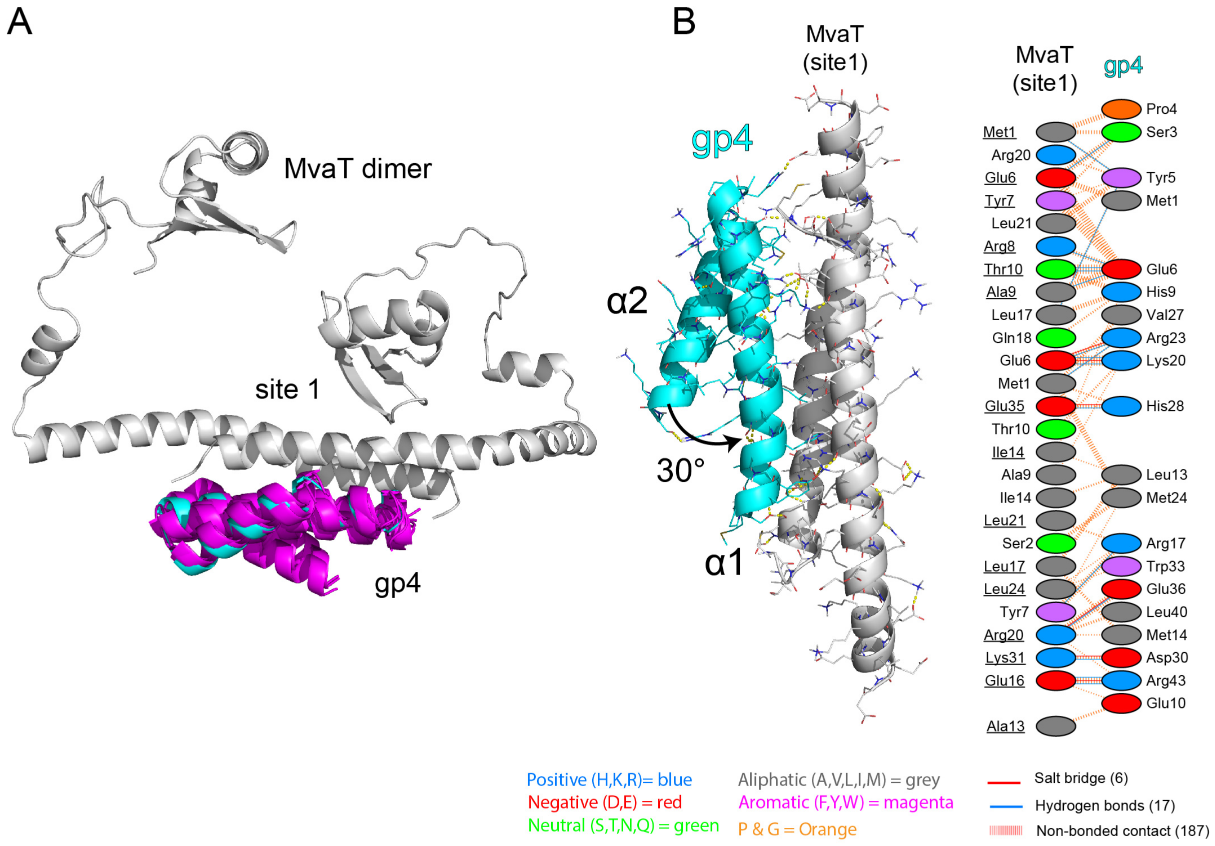


**Figure S3:** (A) HADDOCK best cluster of gp4-MvaT dimer complex. The different orientations of gp4 are shown in magenta cartoon and the MvaT dimer in grey cartoon. The lowest energy structure of gp4 is shown is cyan cartoon. (B) The trimeric coiled-coil complex between gp4 $\alpha1$ and the two helices of MvaT site 1 is depicted in cartoon. The angle between gp4 helices is indicated. The right panel is for the analysis of the intermolecular interactions within the trimeric coiled-coil by PDBsum (1). The underlined amino acids belong to the second helix of the MvaT dimerization site1. The type and number of the intermolecular interaction are indicated in different colors. The amino acids residues of the complex interface are colored based on the chemical characteristics of their side chains, as indicated.

**Table S1.** Structural statistics over 10 lowest-energy NMR structures of gp4. [a]Root-mean-square deviation from the lowest-energy structure calculated for residues 4-45. [b]Calculated with MolProbity.

| | |
|---|---|
| Distance restraints | |
| Total | 1290 |
| Short-range, $\|i - j\| \leq 1$ | 665 |
| Medium-range, $1 < \|i - j\| < 5$ | 406 |
| Long-range, $\|i - j\| \geq 5$ | 219 |
| Dihedral angle restraints | |
| $\varphi$ and $\psi$ | 78 |
| Restraints violations | |
| NOE, > 0.5 Å | 0 |
| Dihedral angle, > 5° | 0 |
| Coordinate RMSD,[a] Å | |
| Backbone | 0.32 ± 0.08 |
| Heavy atoms | 1.09 ± 0.16 |
| Ramachandran statistics,[b] % | |
| Favoured | 99.3 |
| Allowed | 0.7 |
| Outliers | 0.0 |

**Table S2.** Statistics of the top cluster of HADDOCK docking.

| **Cluster 3** | |
|---|---|
| HADDOCK score | -103.3 +/- 14.3 |
| Cluster size | 11 |
| RMSD from the overall lowest-energy structure | 1.0 +/- 0.6 |
| Van der Waals energy | 67.0 +/- 7.6 |
| Electrostatic energy | -360.2 +/- 63.5 |
| Desolvation energy | -5.0 +/- 7.0 |
| Restraints violation energy | 407.9 +/- 78.85 |
| Buried Surface Area | 2369.2 +/- 90.2 |
| Z-Score | -2.0 |

**References:**

1. Laskowski, R.A., Jabłońska, J., Pravda, L., Vařeková, R.S. and Thornton, J.M. (2018) PDBsum: Structural summaries of PDB entries. Protein science, 27, 129-134.
